# Eugenol isolated from supercritical fluid extract of *Ocimum sanctum*: a potent inhibitor of DENV-2

**DOI:** 10.1186/s13568-023-01607-x

**Published:** 2023-10-02

**Authors:** Sulochana Kaushik, Samander Kaushik, Lalit Dar, Jaya Parkash Yadav

**Affiliations:** 1 Department of Genetics, Maharshi Dayanand University, Rohtak, 124001 Haryana India; 2 Centres for Biotechnology, Maharshi Dayanand University, Rohtak, 124001 Haryana India; 3 Department of Microbiology, All India Institute of Medical Sciences, Delhi, 110029 India; 4 Vice Chancellor, Indira Gandhi University Meerpur, Rewari, 122502 Haryana India

**Keywords:** Supercritical extraction, Medicinal plant, Eugenol, qPCR, Antidengue, Docking

## Abstract

Dengue is one of the fairly prevalent viral infections at the world level transmitted through mosquitoes (*Aedes aegypti* and *Aedes albopictus*). Due to various environmental factors, dengue cases surged rapidly at the global level in recent decades, with 193245 cases in 2021 and an increment of 110473 cases in 2022. There is no antidote available against dengue and other *flaviviruses*. In the absence of a dengue vaccine or specific antiviral, medicinal plants or their products can be the only choice for its effective management. *Ocimum sanctum* is known as ‘‘The Incomparable One,’’ ‘‘Mother Medicine of Nature’’ and ‘‘Queen of Herbs’’ in Ayurveda, and is considered an "elixir of life" supreme in both healthcare and spiritual terms. In present study eugenol was isolated in *O.sanctum.* Eugenol (1-hydroxy-2-methoxy-4-allylbenzene) has been substantially responsible for its therapeutic potential. High-performance thin-layer chromatography, Fourier transform infrared spectroscopy and ultraviolet–visible spectroscopy were applied to identify the compound. The Rf value of isolated compound was same in the chromatogram (0.69 + 0.05) with compare to standard. The safe dose of plant and eugenol were found as < 31.25 μg/ml and < 15.62 µg/ml. The anti-dengue activity was assessed in C6/36 cell lines, their effect was determined through Quantitative PCR. The NMR of the isolated eugenol showed similar properties as the commercial marker compound. The eugenol and SFE extract of *O. sanctum* showed the inhibition of 99.28% and completely against Dengue-2, respectively. Docking study exposed that the interaction of eugenol with NS1 and NS5 dengue protein showed the binding energy as − 5.33 and − 5.75 kcal/mol, respectively. The eugenol from the *O. sanctum* plant has the potential to be a good source of future treatment medications for dengue illness, as well as a valuable tool in its successful management

## Key Points

- The optimal yield of the supercritical fluid extraction extract was 0.13 g/10 g (1.3% w/w) at 150 bar pressure
- 40 °C temperature and Ocimum sanctum extract and isolated Eugenol significantly inhibited the DENV-2 virus at a concentration of < 31.25 μg/ml & < 15.62 µg/ml. The isolated extract was identified as Eugenol
- In silico study confirmed the binding energy of ligand with dengue as − 5.33 and − 5.75 kcal/mol, respectively

## Introduction

Mosquitoes are very famous insects responsible for spreading various viral infections in humans. Dengue virus (DENV) is a very notorious virus, belonging to the family *Flaviviridae,* which is a large viral family containing at least seventy-two (72) viruses, out of that thirty-four (34) are mosquito-borne, seventeen (17) are ticks-borne whereas twenty-one (21) are without any known vector (Guarner and Hale 2019; Monath 1994). Dengue virus is transferred by the *A. aegypti* and *A. albopictus* species of mosquito. It is endemic in about 100 countries from Asia, Africa, European and the United States of America. Dengue can be mild to moderate or life-threatening in some cases. Dengue is responsible for 390 million infections out of which 96 million show clinical symptoms annually. In India in 2021 (193245 cases and 346 death) and in 2022 (110473 cases and 86 death) for dengue cases (NVBDCP 2017). Vaccines could be the best possible treatment for viruses, dengue vaccines against all four serotypes is a crucial problem due to antibody-dependent enhancement (ADE) (WHO 2020). WHO considers that Ayurveda medicine extracted from medicinal plants is generally more secure, easy to find anywhere, non-toxic, comparatively less perilous and inexpensive than synthetic drugs. For antiviral we are looking toward natural products, which are considered to be non-narcotic, have negligible side effects, and are economical. Herbal products are an important part of health care during the whole journey of our development (Kaushik et al. 2018).

*Ocimum sanctum* (L.) relation to the *Lamiaceae* family and is a renowned traditional Ayurvedic herb. It is popularly known as Holy Basil (English) and Vishnu-Priya, Tulsi (Hindi). It is a seasonal plant found throughout India. The growing conditions are moist to wet, rich loamy well-drained soil, grown in warm and saline to acidic alkaline soils suitable for cultivation. The herb is aromatic, erect, and branched, about 75 cm in height. It is cultivated at spiritual places such as temples and also in houses for routine worshipped by Hindus. It contains many pharmacological properties (Nagarajan et al. 2022; Siva et al. 2016). The extract of *O. sanctum* contains many flavonoids like orientin, vicenin, luteolin, tannin, saponins, carbohydrates and protein. The two components eugenol and ursolic acid are very useful for antibacterial and antiviral purposes (Mittal et al. 2018; Kirtikar and Basu 1999). *Ocimum sanctum* extract was prepared by SFE machine. Under specific temperatures and pressures, the SFE extractor machine can isolate specific compounds from a mixture of medicinal plant extract. In the SFE machine, carbon dioxide (CO_2_) is employed as a solvent. CO_2_ is is non-toxic, clean, non-flammable, low-cost gas and its temperature (31.1 °C) and pressure (72 bar) are relatively low and easy to handle. The herbal compounds help to reduce many types of diseases. In the present study, Eugenol (1-hydroxy-2-methoxy-4-allylbenzene) has been isolated from *O. sanctum* SFE extract and characterized with different techniques, and both extract and isolated compound tested against the dengue-2 virus in vitro.

## Materials and methods

### Plant collection site

*Ocimum sanctum L.* was selected on the basis of its ethnobotanical applications. The plants were grown in the M. D. University botanical garden in Rohtak, India (Latitude: 28°53′24.97′′N and Longitude: 76°34′57.252′′E).. Experimental plant was identified using taxonomic keys compared to voucher no. D.DUN 25417, and the specimen was deposited to M.D.U Herbarium Rohtak part of Haryana, India with voucher no MDU5804. *O. sanctum mature*, clean leaves were taken from plants that were fully developed to maturity. To eliminate the dust, the collected leaves were rinsed with clean tap water and then properly wash with double-distilled water. The washed leaves of *O. sanctum* were incubated for air-dried at lab-temperature in the shade before being pulverized in a mixer grinder.

### Employing a supercritical fluid extraction (SCFE) process to make the *O. sanctum* extract

The extraction Machine (Applied Separation Inc.U.S. SFE Prime) was employed to prepare an extract of the *O. sanctum*. 10 g of fine plant fine particles were packed into the stainless steel cup attached to the extractor in the machine. CO_2_ was used as a solvent, their flow rates adjust in the range of 1.8 ml/min in static-dynamic mode (1 h static and 40 min dynamic mode).The *O. sanctum* SFE was collected in a collecting tube. To get rid of any CO_2_ residues, the extract was incubated at room temperature. The *O. sanctum* SFE process was standardized and optimized (Table 1) for the separation of specific secondary metabolites from the crude extract. Lyophilization was performed of the extracted plant extracts was done by the Lypholizer (Hyper Cool HC3110, Hanil Scientific Inc.). The dried *O. sanctum* extract was weigh and kept at 4 °C till further use. Following calculations were made to determine the percentage yields of *O. sanctum* extracts: % age yield is calculated as follows: Weight of the extracted material divided by weight of the dried material multiplied by 100.

**Table 1:** Yield gained from *O. sanctum* SFE extract

Temp	Sample	Applied Press	Yield	% of the yields %
40 °C	10 g powder	10 MPa	0.11 g	1.1
40 °C	10 g powder	15 MPa	0.13 g	1.3
40 °C	10 g powder	20 MPa	0.10 g	1.0
40 °C	10 g powder	25 MPa	0.066 g	0.66

### From *Ocimum sanctum* SCFE extract compound identification

The supercritical extract was isolated from 10 g dry powder of *O. sanctum.* The desired component of SFE was identified by HP-TLC along with a commercially available marker compound (eugenol). Hydrogen & Carbon Nuclear Magnetic Resonance (1H NMR and 13C NMR), Fourier-Transform Infra-Red Spectroscopy (FT-IR), and Ultraviolet–Visible Spectroscopy were used to further characterise isolated Eugenol (1-hydroxy-2-methoxy-4-allylbenzene) from the SCF extract.

### HPTLC (High-performance thin layer chromatography) examination

The marker eugenol (> 99.8%) was acquired from commercial Sigma Aldrich (India) and diluted in varied amounts of methanol (HPTLC grade) (2 μl, 4 μl, 6 μl, 8 μl and 10 μl). For HPTLC, an SFE stock of *O. sanctum* was set as 10 mg extract/ml methanol. The mobile phase for eugenol separation was prepared (Toluene: ethyl acetate: formic acid (90: 10: 01, v/v) (Khan and Ali 2014). The mixed investigation sample were filter through 0.25 m membrane filter (MILLEX^®^ GV) before being placed to silica plate (10 × 10 cm, 60 F254, E. Merck) and heated at 100 °C for 10 min. The different dilutions of the standards (2 μl, 4 μl, 6 μl, 8 μl and 10 μl) were apply on TLC plates, 10 mm on top of from the bottom line with the help of CAMAG automatic sample loading applicator all along with N_2_ flow (Lino mat V). 5 µl of SFE extract sample at various concentrations was put to TLC plates in the outline of 5 mm wide bands with the help of a Hamilton microsyringe (100 µl). The TLC plate was photographed in the 200–600 nm range of visible and UV light. The retained factor (Rf) value was calculated using the resolved spot, which was then compared to the Rf value of the commercially used marker (eugenol). The eugenol band was identified with the help of a commercial marker (eugenol) band. The band was scraped and dissolved in one milliliter of aqueous solution. After removing the silica, the eugenol was collected into 2 ml vials and lyophilized.

### Proton and carbon nuclear magnetic resonance spectroscopy (1H NMR and 13C NMR)

The spectra was obtained in Deuterated chloroform (Cdcl_3_) using a Bruker Avance III, 400 MHz (Agilent, USA). The value of chemical shifts are reported in the ppm. In the NMR spectra, tetra-methylsilane (TMS) was employed as an internal standard.

### Isolation of viruses and culture of C6/36 cells

For dengue viruses, the C6/36 a mosquito-derived cell line has been recommended. NCCS Pune provided C6/36 (ATCC^®^ CRL-1660 TM). The cell line was maintained prescribe MEM media supplemented by 10% Fetal Bovine Serum (FBS), 2mML-glutamine, penicillin (100 U/ml), and streptomycin (100 g/ml) by following the standardized animal cell culture protocol. Cells were cultured at 28 degrees Celsius in a humidify environment with 5% CO_2_ (Acosta et al. 2011). The media were changed according to the situation. In a 25-T cm^2^ tissue culture flask, 500 µl of an adequate dilution of dengue-2 viral standard-strain was introduced into C6/36 cells. In our prior study, we described the viral isolation process in detail (Kaushik et al. 2021a).

### A stock preparation of *O. sanctum* for cell viability

On the basis of solubility, stock solutions of extracted eugenol were obtained by dissolving 1000 µg extract/1 ml in culture media (minimum essential medium), and the resultant solutions were serially diluted to concentrations ranging from 500 µg/ml to 3.9 µg/ml. The pH of the cell culture medium used for dissolved was kept constant at pH 7.0. 0.22 m syringe filter (MILLEX^®^ GV) was used to sterilise the dissolved extract. Stocks of extracted plant material were kept at − 20 °C for future use.

### In C6/36 cells, the maximum non-toxic dosage (MNTD) of *O. sanctum* SFE & commercial eugenol was determined

The MTT test was employed to determine the maximum non-toxic dose. 1.2 × 10^6^ cells/well C6/36 cells were added onto a flat-bottom ninty six plates (Nunc, Thermo Fisher Scientific, USA). In brief, after 80% of the cell confluency levels, they were treated with varied doses (1500 µg/ml to 23.43 µg/ml) of *O. sanctum* SFE and commercial eugenol in triplicate. The inoculated plate was incubated at 28 °C in a 5% CO_2_ incubator for a period overnight. A cell control (medium-plated cells) and blank-control (medium) were also included in the experiment. The detailed procedure for MNTD has followed the same procedure as in our previous study (Kaushik et al. 2020, 2021b). Afterward, absorbance values of *O. sanctum* SFE and commercial eugenol were noted with the help of a microplate reader (Bio-Rad, USA). Absorbance readings made at 595 nm using a microplate reader (Bio-Rad, USA) were used to calculate the vitality of the cells.

### Anti-dengue assay in vitro

The anti-dengue experiment was carried out on an 80% confluent monolayer of C6/36 cells in 96-well culture plates. The experiment was done in conjunction with a cell-control (Negative-control), a virus-control (Positive-Control), and back-titration. The experiment was carried out with 100copies of the standard strain of the dengue-2 virus. The cell layer could be damaged during the experiment, so a replica plate was prepared. In a replica plate, 100copies/ well of dengue-2-virus suspension were pre-treated with non-toxic plant extracts of *O. sanctum* SFE and commercial eugenol for 60 min.The replica plate was softly shaking in every 10 min.The material from the replica plate was gently transferred into respective wells of the experimental plate without disturbing the cell layer. The details of in vitro assay and RNA extraction are given in our previous paper (Kaushik et al. 2021a).

### qPCR assay

The inhibition effect or value of *O. sanctum* SFE and commercial eugenol against the virus of DENV2 was determined with the help of a qPCR. Unfortunately, Dengue does not produce a cytopathic effect or morphological changes, therefore can not be compared through the reduction of the TCID_50_ or plaque assays (Azarkh and Robinson 2008; Lambeth et al. 2005; Bente and Rico-Hesse 2006). qPCR is a more advanced technique that gives quantitative results. The virus titers of dengue-2 were confirmed by using commercial kit Geno-Sen’s. The kit has been supplied with a known copy number of dengue standards (positive control). The kit contained different concentrations of dengue (10^1^, 10^2^, 10^3^, 10^4^, and 10^5^)copies/ml. A standard calibration curve for the dengue virus was created using a known copy number supplied in the kit. The copy number of dengue viruses in cell culture lysate was estimated by comparing the calibration curve produced by qPCR to a known copy number standard provided in the kit. Through serial dilution, the desired copy number for further experimentation can be obtained from the lysate. The present study is following the same protocol as our previous study (Kaushik et al. 2021a).

### Data analysis

Tukey’s test was performed to calculate the maximal non-toxic dosage of the *O. sanctum* SFE and isolated eugenol based on the cell viability or toxicity percentages. The cell viability was compared between positive, and negative controls as well as with each other. All samples were tested in the triplicate formate. GraphPad PRISM for Windows 5 (Software Inc., San Diego, CA, 2005)/Microsoft Excel 2007 was employed for statistical analyses.

### Preparation of ligands and proteins structure

NS1 is well known for harmful activities responsible for serum leakage and affecting coagulation may result in more severe dengue (Lengauer and Rarey 1996). NS5 is the biggest protein identified in flavivirus genomes. NS5 is the biggest and most drug-targeted domain in the Flaviviruses. To govern viral replication, NS5 comprises methyltransferase and RNA-dependent RNA polymerase (RdRp). NS5 is responsible controls the RNA splicing during dengue replication at the 5′UTR within in the host cell and inhibits the host immunological response and interferon action. (Tahir et al. 2019). The 3D structures of the ligand eugenol were obtained from PubChem (PubChem IDs: 3314). Using Chimera’s Dockprep module, structures were then minimised and made dockable. The 3D structure of the DENV-2 virus non-structural proteins NS1 (PDB IDs: 4O6B) and NS5 (PDB IDs: 4V0Q) were determined using PDB (protein data bank). Bound prosthetic groups such as NAG, acetate ion, Zn, and Glycerol were removed. The docking investigation between NS1 and NS5 proteins of dengue and the specified ligand eugenol was performed using the Auto-dock program (V4.2.6 Protein). It was prepared for docking study by combining polar hydrogen with carbon and adding Kollman charges. To accomplish blind docking, the Grid box was expanded to the whole protein with a spacing of 0.375. The ligand was created by controlling the overall number of torsions accessible, resulting in a more flexible molecule. The docking search was carried out using a Lamarckian Genetic Algorithm (4.2). The docking complex was saved using the MGL tool, and the interaction was visualised using Lig Plot Plus.

## Results

### Optimum extraction condition

The various parameter for SCFE extraction conditions were applied for isolating particular components from *O. sanctum* crude extract. During optimization, it was found at 40 °C temperature and 15 MPa pressure give the best result. From the optimized condition maximum amount of *O. sanctum* SFE extract was 0.13 g/10 g (1.3%w/w/10 g test sample) obtained (Table 1). Supercritical fluid extraction (SCFE) was selected or standardized on the basis of the yield. various advanced analytical techniques were applied for separating and analyzed the desired compound from the SFE of *O. sanctum*. The composition of SFE metabolites may be changed when the temperature and pressure parameters change. The best extraction conditions of plants were standardized on the basis of higher yields obtained from the SFE plant extract.

### Maximum non-toxic dose of plant extract

**Fig. 1:**
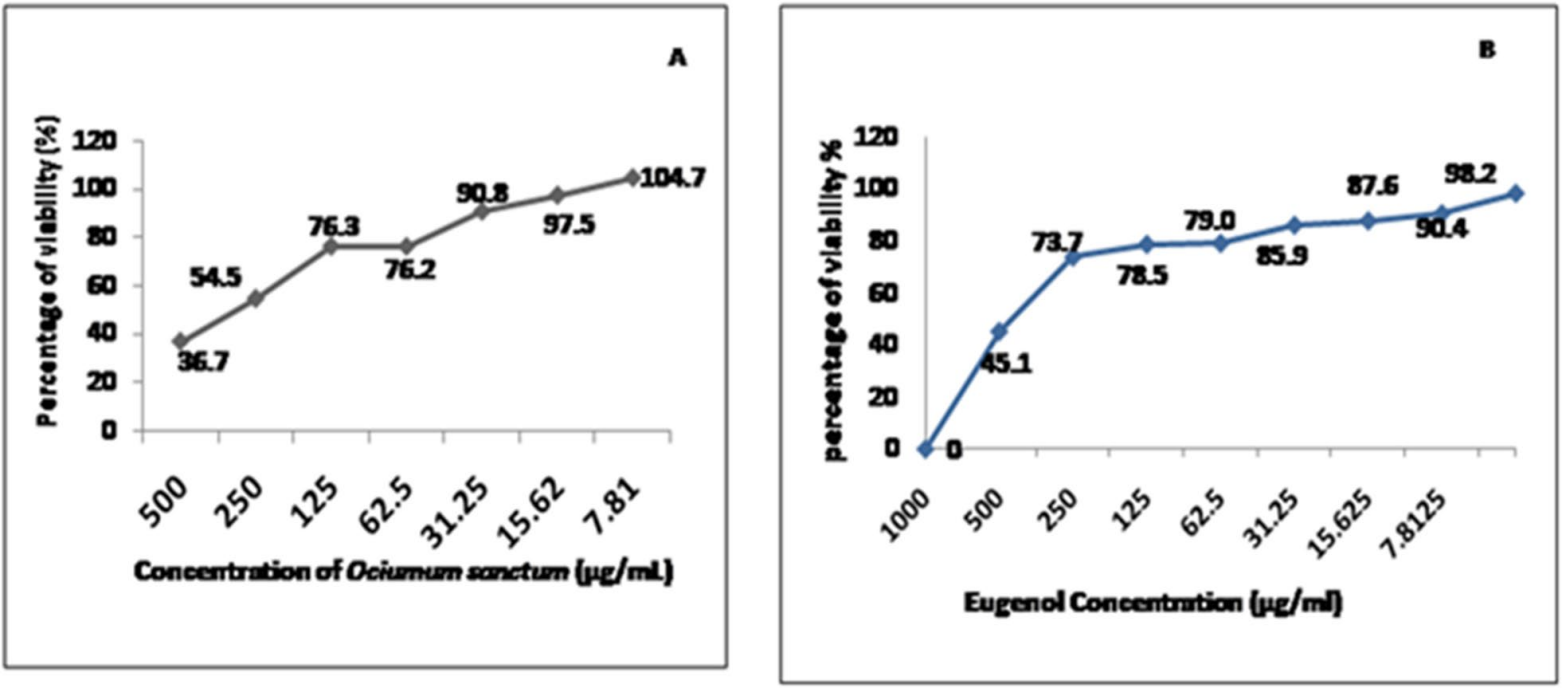
**A** In cell-line test, the maximum non-toxic dosage (MNTD) of *O. sanctum* SFE extract) **B**, MNTD of isolated Eugenol

The maximal non-toxic doses of *O. sanctum* SFE extract and isolated eugenol from *O. sanctum* SFE extract by HPTLC and were determined to be 31.25 µg/ml (Fig. 1A) and 15.62 µg/ml (Fig. 1B), respectively. The MNTD was determined based on the cell-viability of the mosquito cell lines (C6/36) followed by the MTT assay. The result suggests that both the *O. sanctum* plant SFE and isolated eugenol at concentrations of < 31.25 μg/ml and < 15.62 µg/ml are non-cytotoxic.

### FTIR superimposed figure of the isolated SFE of *O. sanctum* and commercial eugenol (Marker)

**Fig. 2:**
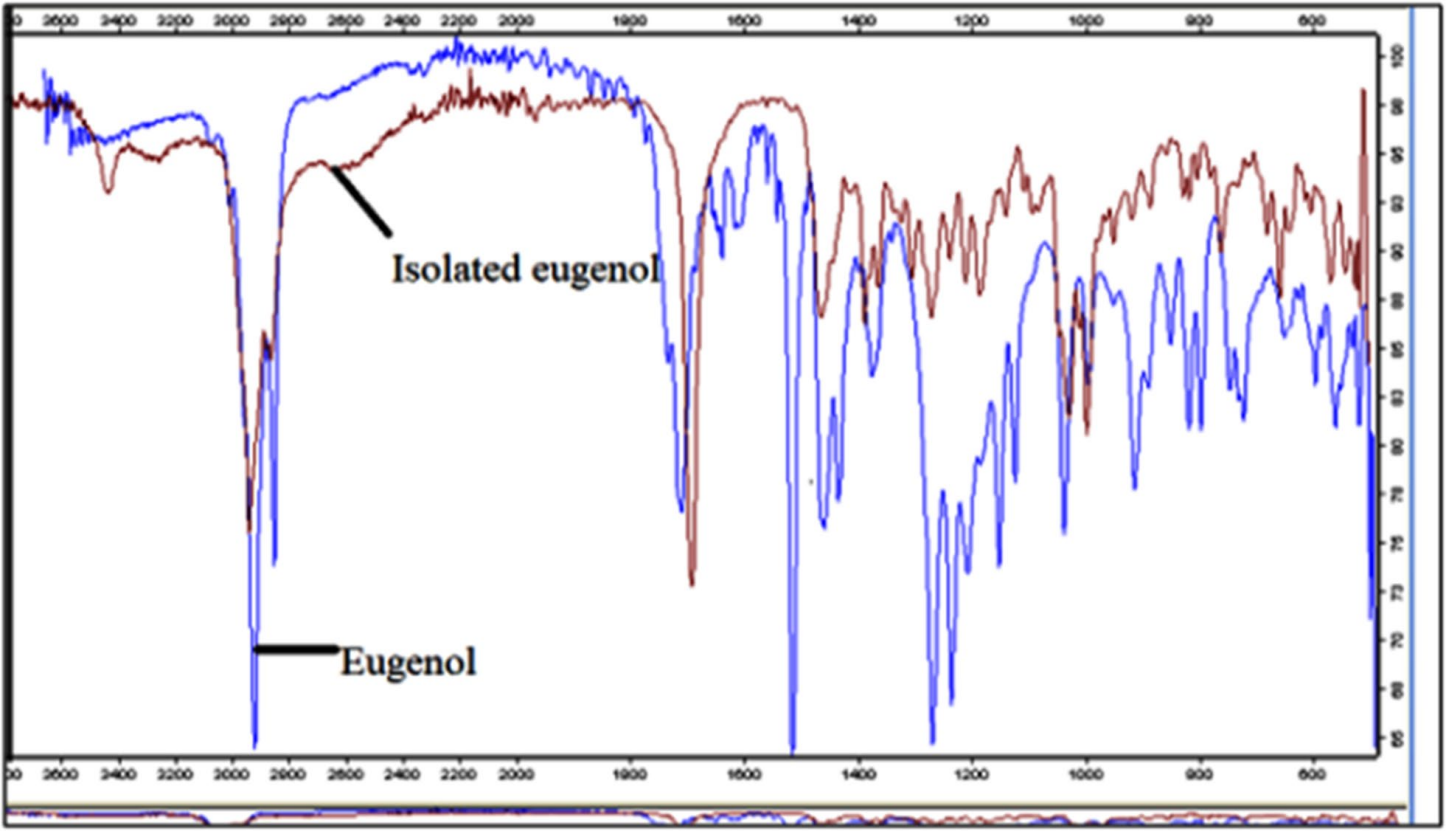
FTIR superimposed figure of the isolated SFE of *O. sanctum* and commercial eugenol (Marker)

The FT-IR spectra band of isolated eugenol from *O. sanctum* SFE extract were at 3566, 3075, 3006, 2939, 2918, 2839, 2849, 2389, 2371, 2322, 2202, 1998,1 940, 1894, 1708, 1733, 1637, 1610, 1615, 1576, 1558, 1541, 1514, 1458, 1432, 1374, 1267, 1234, 1206, 1150, 1122,1034, 993, 888, 816, 795, 744, 720, 649, 593, 558, 518, 510. The FT-IR band of isolated eugenol displayed sharp bands at 3075 cm^−1^ (-N–H-stretching) and at 3700–3548 cm^−1^ (-O–H- stretching) which were indicators for the occurrence of primary amines and alcohol. The FTIR band of isolated eugenol displayed sharp bands at 3075 cm^−1^ (-N–H-stretching) and at 3700–3548 cm^−1^ (-O–H- stretching) which indicators the occurrence of primary amines and alcohol. The peak at 2850–3006 cm^−1^ (-C-H- stretching) was showing the presence of alkyne. 1400–1600 cm^−1^ (-C–C- stretching) peaks were showing aromatic compounds. The peak at 2918–2389 and 1350–1480 cm^−1^ (-C-H-stretching) centimeter^−1^ in the spectra of FT-IR confirmed the presence of the alkane compounds. The peak at 1000–77-1250 cm^−1^(-C-N-or-C–O–C-stretch) represented the indicators of amine compounds. The band at 1000–1400 cm^−1^ and 600–800 cm^−1^ (-C-F-stretch) & 500–600 cm^−1^ (C–Br stretch) showed the alkyl-halides. The height at 1708 cm^−1^ (-C = O stretch) showed the primary alcohol and peak at 911 cm^−1^. In spectroscopy of FT-IR of both SFE of *O. sanctum* and commercial eugenol, superimposed peaks were obtained at various places. Their superimposed were detected at 3318, 1458, 1234 and 1034 cm^−1^, due to the manifestation of -C–O–C, OCH3, -C-H-, O–H whereas the alkyl group 2939 cm^−1^, in the experimental and standard samples. Hence, FT-IR spectra revealed the extracted compound from the *O. sanctum* was eugenol. Although, the sharp peak at 3518, 2939, 1637, 1615, 1514 cm^−1^, 1458, 1234 and around 1034 were well in agreement with the FT-IR spectrum of pure eugenol which represented the presence of eugenol in supercritical extracts of *O. sanctum* in Fig. 2.

### UV spectroscopy of isolated SFE of *O. sanctum* and commercial eugenol

**Fig. 3:**
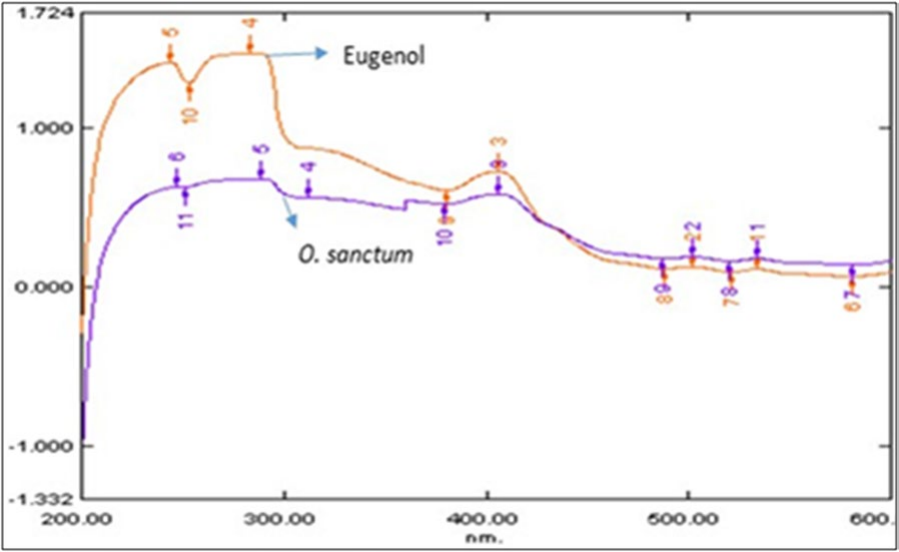
Overlay spectra of isolated Eugenol in *O. sanctum* SFE with standard

The identification of eugenol in plant extract was confirmed by overlapping the UV absorption spectra of the plant extract sample & with the markers compound. Broadband was observed at 280 nm in marker eugenol (Fig. 3). The peaks of the plant sample and the spectrum of the respective marker were found to be overlapped at a similar wavelength. This shows that the isolated component from *O. sanctum* SFE extract is eugenol, as it exhibits characteristics comparable to commercial standard eugenol employed as a marker.

### HPTLC examination

**Fig. 4:**
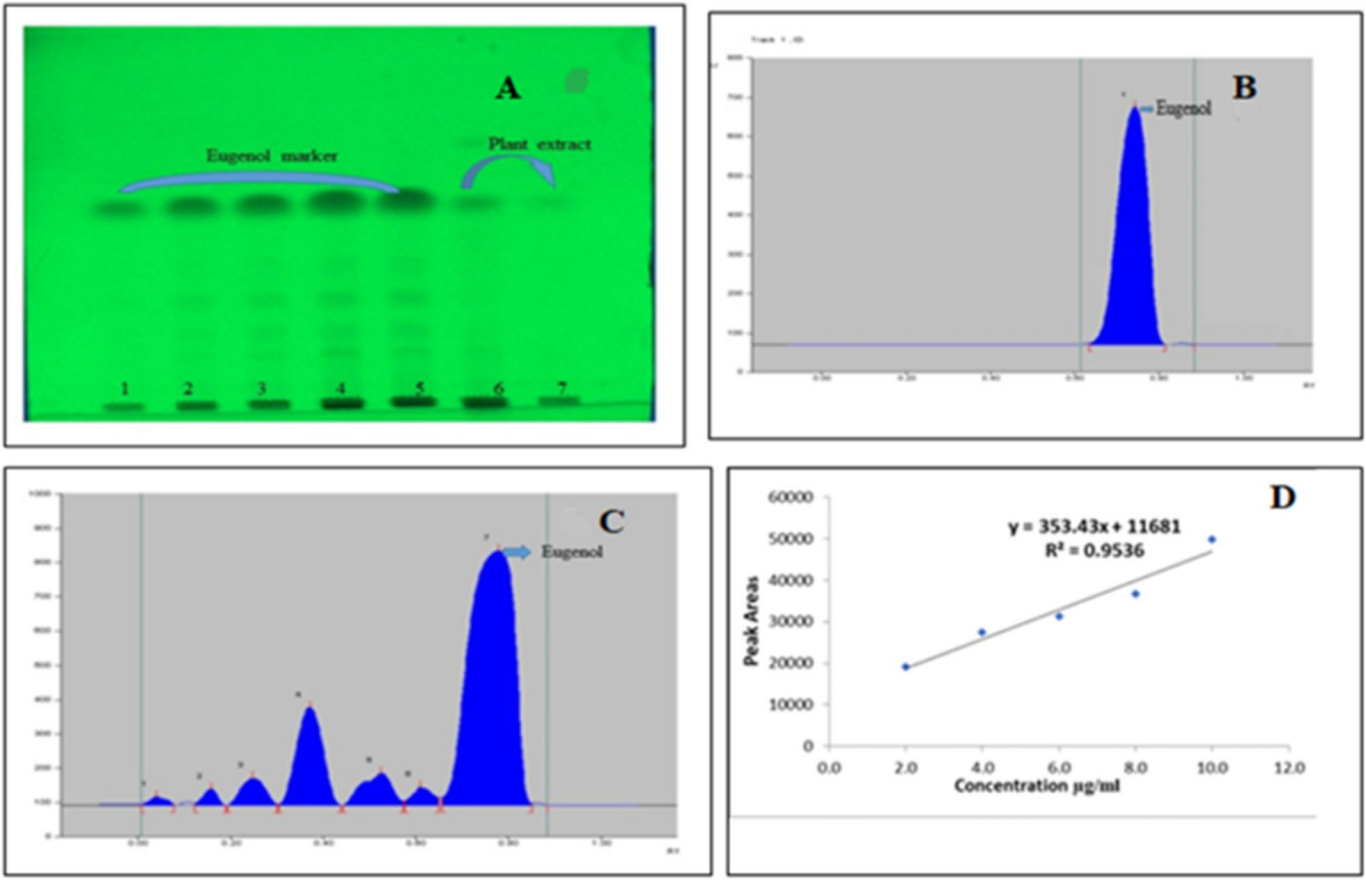
**A** TLC with marker compound (1-5position) plant extract (6to7position), **B** Marker eugenol, **C** Densitogram of *Ocimum sanctum* SFE), **D** Calibration curve

The existence of eugenol in the test sample was verified by HPTLC analysis, which showed the similar Rf value of extracted & marker compound in the chromatogram (Rf 0.69 ± 0.05), and the eugenol compound was extracted from the prepared TLC plates in a structure identical to eugenol. The band that appears due to the eugenol was confirmed based on a comparison with the band commercial marker band on the TLC plate in triplicates (Fig. 4A). The band was scratched from the TLC plate and dissolved in 1 ml aqueous. The silica was removed from scratched material with the help of centrifugation and this technique repeat several times and then collected into 2 ml vials and lyophilized. The complete HPTLC chromatogram of *O. sanctum* SFE extract by commercial marker compound was given in Fig. 4B, C. The linearity equation of eugenol was developed by regression analysis as a consequence of the calibration curve, and the results revealed a satisfactory linear connection (2.0–10.0 g/spot) (Fig. 4D). The R^2^ value of eugenol was 0.9536. This R^2^ value signifies how close the data fit the regression line. The linear regression line was used to calculate the concentration of eugenol in the selected experimental crude of tested plant. In the prior study, this method was validated by recovery, precision, robustness, accuracy, and reproducibility (Khan and Ali 2014).

### Estimation of eugenol in herbal extracts

The linear regression lines were used to calculate the amount of eugenol in the experimental sample (Y = 353.43x + 11,681). 16 µg/ml concentration was available in the 50 µg crude SFE of the *O. sanctum.* The average yield of the eugenol content in the *O. sanctum* supercritical extract was determined to be 32%w/w. 16 µg/ml concentration is available in the 50 µg crude SFE of *O. sanctum.*

### Carbon nuclear magnetic resonance (13C NMR) and proton nuclear magnetic resonance (1H NMR) (of eugenol)

**Fig. 5:**
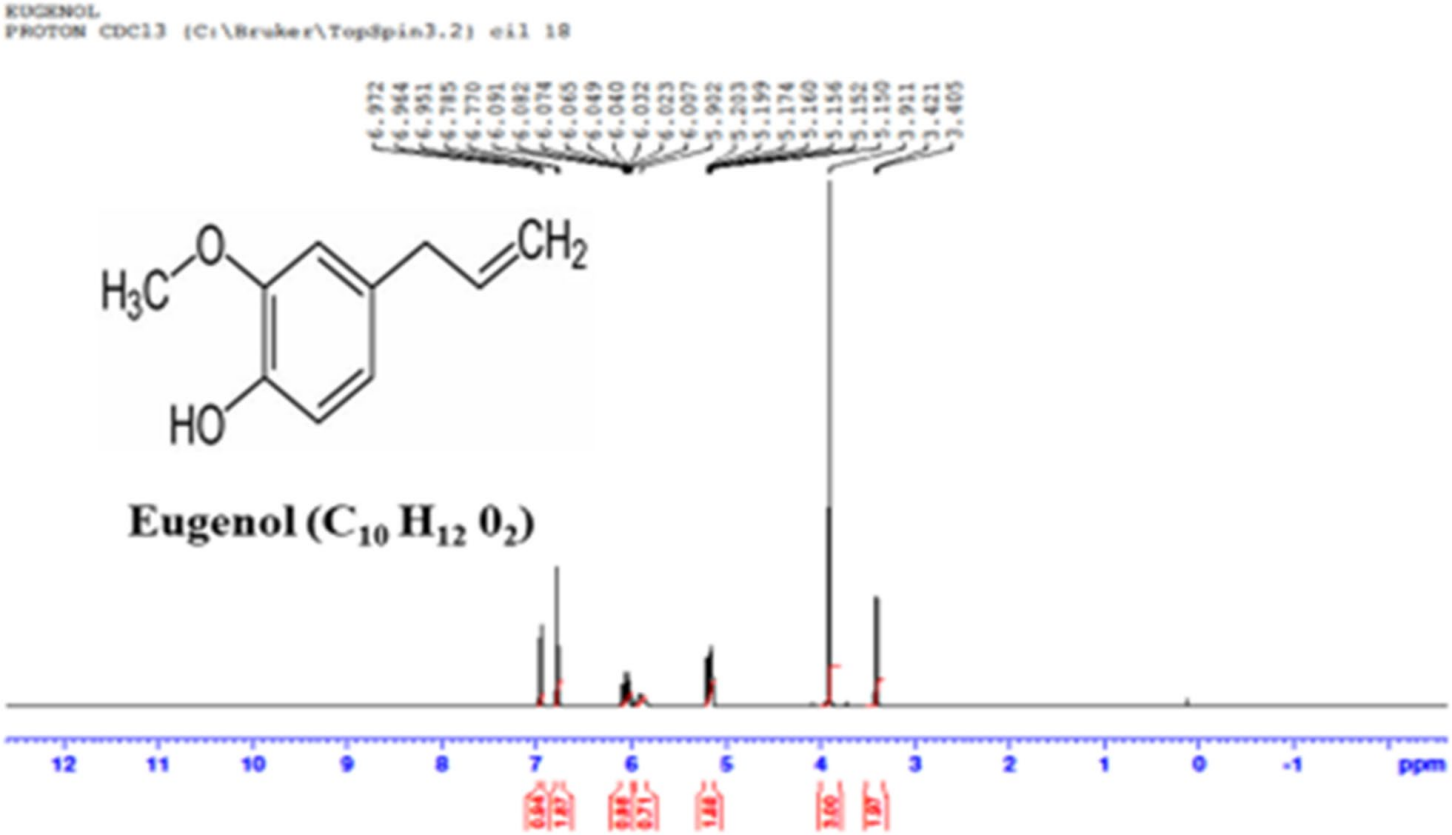
1H NMR of isolated SFE of *O. sanctum* (Eugenol)

**Fig. 6:**
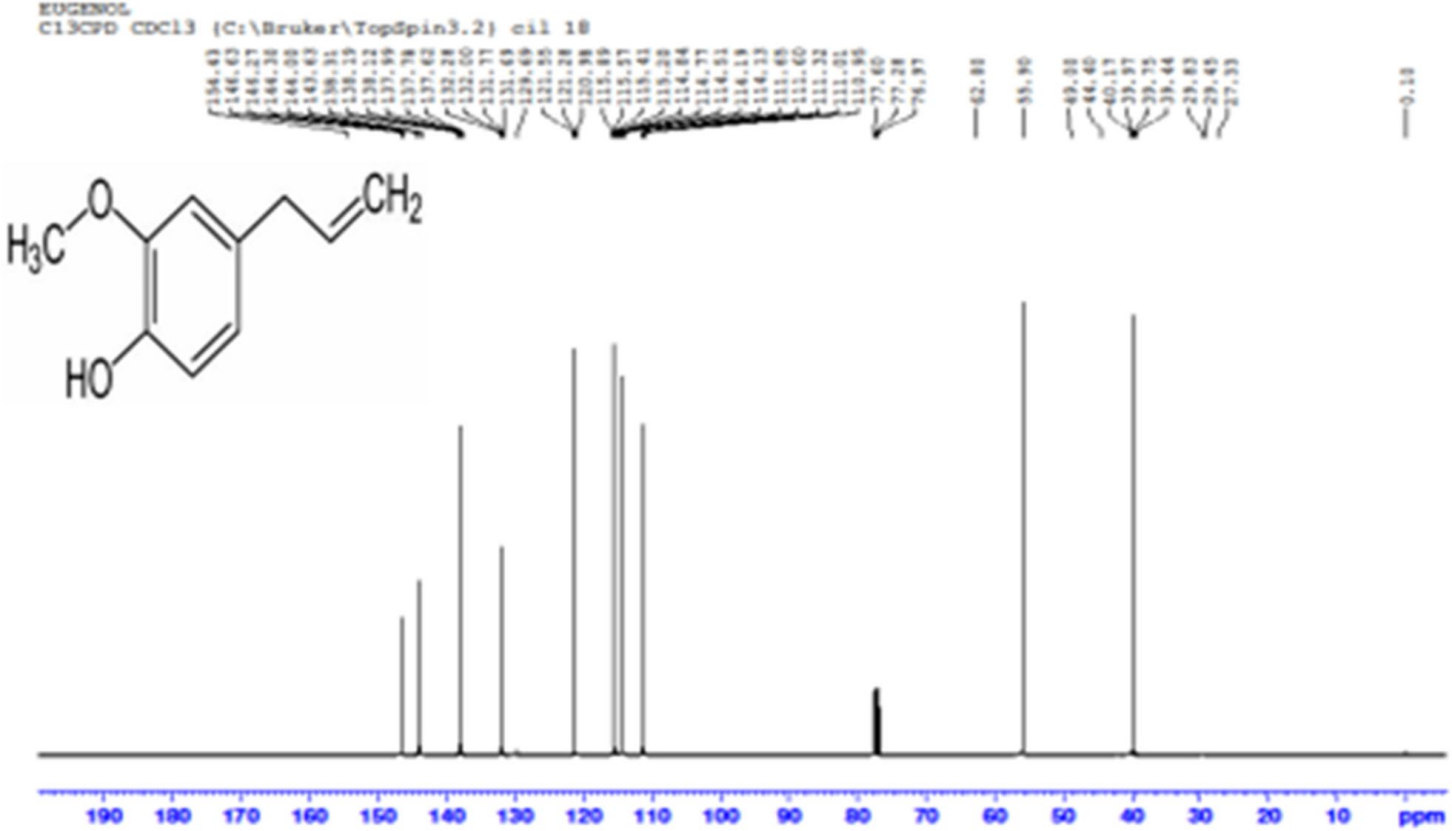
13C NMR of isolated SFE of *O. sanctum* (Eugenol)

1H NMR -(CDCL3): 3.21 (d, 2H), 3.81 (s, 3H), nearby 3.84 (s, 2H), 3.97 (s, 2H), 4.88 (s, 2H), 5.04 (d, 2H), 5.87 (m, 1H), 6.25–6.84 (m, 4H), 7.29 (d, 1H) Fig. 5. 13C NMR- (CDCL3, PPM): 39,47,48,55,81,108,109,114,115,118,120,131,136,139,141, 146,152; m/e value. The results data of 1H NMR and 13C NMR resemble eugenol structure Fig. 6.

### qPCR assay

**Fig. 7:**
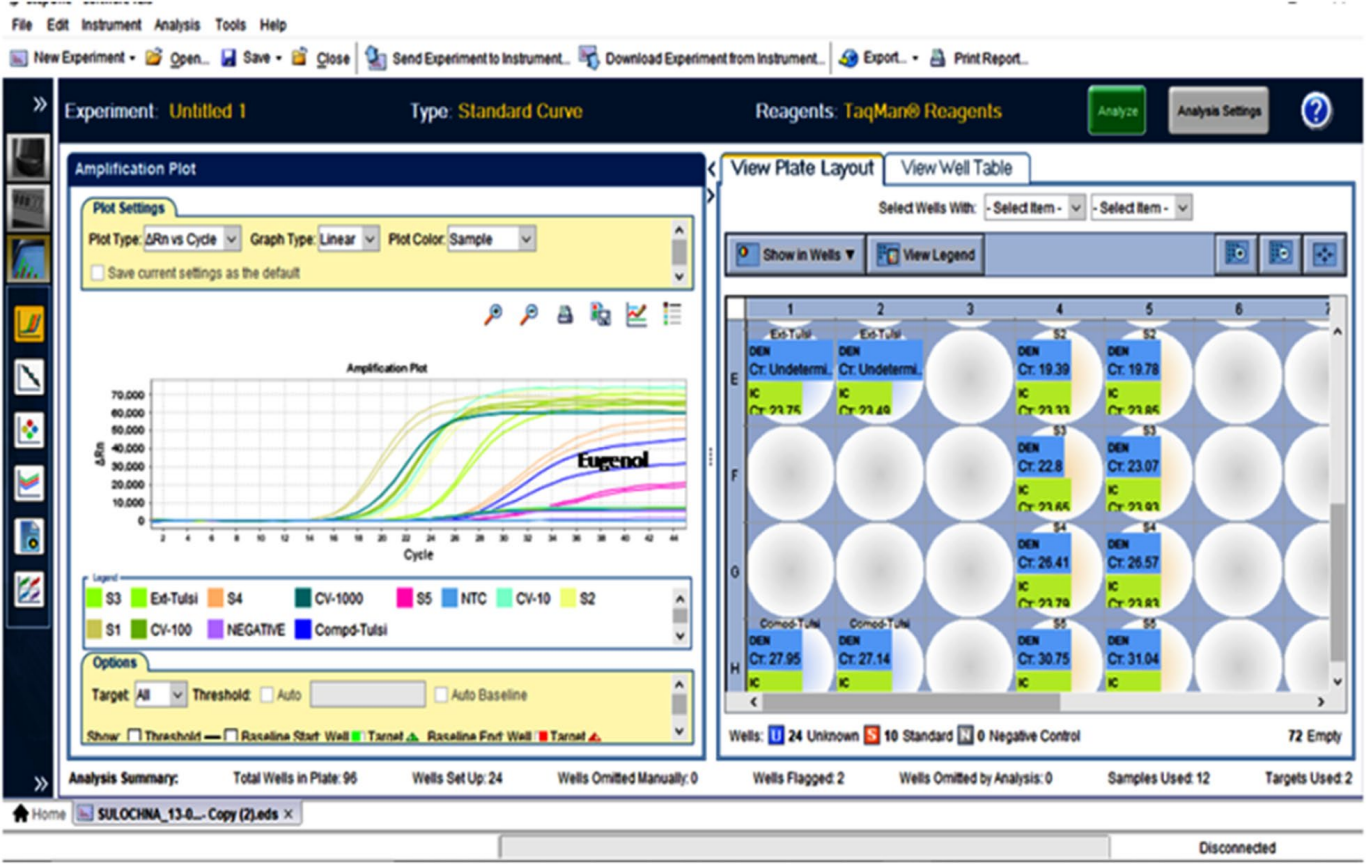
Representation of dengue viral inhibition by *Ocimum sanctum* SFE extract and isolated Eugenol amplification curve: (S1 to S5 are positive vius control; CV-10, CV-100 and CV-1000 copies/ml; Tulsi (*O.sanctum* extract); Blue- Eugenol isolated compound from Tulsi

The current study demonstrated that the *O. sanctum* supercritical extract and commercial eugenol significantly inhibited dengue. The *Ocimum sanctum* plant SFE extract showed complete inhibition as follows as isolated eugenol showed 99.28%, treated with a concentration of 31.25 μg/ml (MNTD) and 15.62 μg/ml, respectively against Dengue-2. Table 2 displays the results of the anti-dengue activity of eugenol as determined by qRT-PCR. The results were compared with positive control of dengue-2 serotype copies number or CT value. As a result the *O.sanctum* was completely inhibited the dengue virus and the extracted Eugenol inhibited as 99.28%. The resulting amplification curve by qPCR depicting the anti-dengue potential effects of *O. sanctum* SFE extract and commercial eugenol is shown in (Fig. 7). After 7 days of post-infection, a copy number of 2.80 × 10^6^ copies/ml was detected in the virus control of the isolated eugenol.

**Table 2:** Anti-DENV-2, activity of isolated eugenol from *O. sanctum* supercritical extract

Sr. No	Compound name	Quantity mean values (virus copies/ml)	Quantity (SD value)	CT Mean	CT SD (Value)	% Compound inhibition^a^
1	Eugenol	20,167.95	6096.60	26.61	0.55	99.28%
2	Viruspositive control(100copies/ml)	2808999.0	102,602.78	17.68	0.07	–

^a^% inhibition = mean of examination sample/mean of viral control cells) × 100

### Eugenol docking with dengue NS1 and NS5 protein

**Fig. 8:**
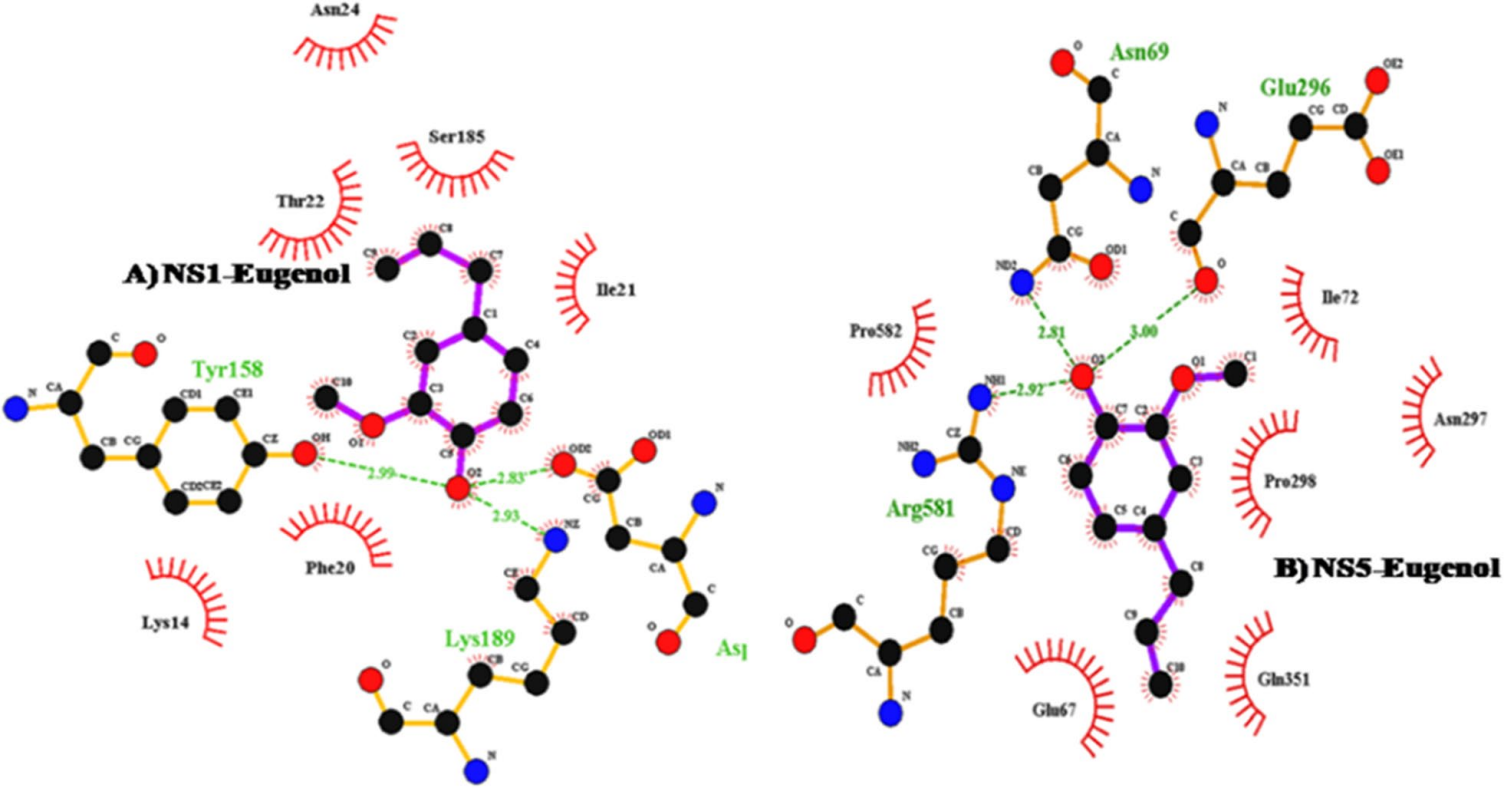
**A** Ligand plot of amino acids involved in eugenol interactions against the NS1 protein, **B** Ligand plot of amino acids involved in eugenol interactions with the NS5 protein

Overall results indicate that eugenol is present in the SFE of *the Ocimum sanctum.*With a binding energy of -5.33 kcal/mol and H-bond distances of 2.99, 2.93, and 2.83, respectively, eugenol demonstrated three interactions with protein NS1. The inhibition constant value was 44.17 µM, and ele and Vdw interaction are combined within entire binding energy. Ligand Eugenol had 3 putative interactions against the dengue NS5 protein. The binding energy of the interaction between eugenol and NS5 of dengue protein is − 5.75 kcal/mol, and the three residues implicated were Asn69, Glu296 and Arg58, based on the results of molecular docking. The H-bond distance was found 2.81 Å, 3.00 Å and 2.92 Å, respectively. The inhibition constant value was 61.17 µM and Vdw and ele interaction were combined within a total energy -6.76 kcal/mol. The hydrogen bonds & the wander wall interactions in compounds against dengue NS1 and NS5 protein have been shown in ligplots Fig. 8.

## Discussion

There are four serotypes of dengue virus (dengue 1 to 4) which is responsible for dengue in human beings. Dengue-2 serotype was circulated worldwide as compared to other serotypes. No drugs are available in the world only suggestive and supportive care is given to the patients (WHO 2023). Many drugs have been used which is isolated from natural plants against many types of disease (Kaushik et al. 2018; Neelawala et al. 2019). In the current design, the choosen plant (*O. sanctum*) extract was prepared by using a non-conventional method *i.e.* supercritical fluid extraction (SFE CO_2_). The extraction methods were standardized by varied temperatures and pressure on the SFE machine. Various parameters which provide the maximum yield of plant extract was selected. Gosh et al. obtained the eugenol compound at 70 °C temperature and 40 MPa pressure as (Gosh et al. 2013) compared to the present study in which the maximum yield of eugenol was obtained at 15 MPa pressure and 40 °C temperature. The CO_2_ gas was used as a solvent because of its non-flammable nature and relatively low temperature and pressure. The eugenol yield was obtained at 4.63 mg/g from Krishna Tulsi dry powder by SFE extraction. Kaushik et al. obtained a 1.8% w/w yield of andrographolide in SFE extract of *A. paniculata* (Kaushik et al. 2021b). Kaushik et al. obtained yield of *Cyamopsis tetragonoloba* SFE extract is 1.3% (Kaushik et al. 2020) as well as *Berberis vulgaris* (3.5%/10 g), *Phyllanthus niruri* (4.8%/10 g), *Carica papya* (2.5%/10 g) at 60 °C and 200 bar (Kaushik et al. 2021c). *Tinospora cordifolia* (0.9% yield) yield was obtained as well as in the present study *Ocimum sanctum* has 1.3%w/w out of 10 g plant crushed sample. Other studies have demonstrated the anti-dengue virucidal effects of SFE extracts of *Andrographis paniculata* (1.3% yield), *Berberis vulgaris* (3.5%), *Carica papaya* (2.5%), *Cymbopogon citrates* (1.6%), *Euphorbia hirta* (1.3%), *Leucas cephalotes* (1.3%), *Phyllanthus niruri* (4.8%), and *Tinospora cordifolia* (0.9%) as well as others study have already been reported (Kaushik et al. 2021c). In FT-IR analysis, the superimposed peaks were identify at 3318, 1458, 1234 and 1034 cm^−1^, due to the form of -C–O–C, OCH3, -C-H-,O–H whereas the alkyl group 2939 cm^−1^, in experimental sample and standard. The UV overlay spectra and NMR spectra matched the marker eugenol compound. Eugenol is hepatotoxic, so an overdose of eugenol causes a wide range of symptoms i.e. nausea, rapid heartbeat, diarrhoea and oblivion, blood appears in the patient’s urine. So firstly, a non-toxic dose must be required. Tang et al. have reported that the extract of methanol of MNTD of *O. sanctum* was 0.100 mg/ml, against the Dengue serotype1 on Vero E6 cell (Tang et al. 2012). Ling et al. reported that methanolic extracts of *O. sanctum* below the MNTD were 0.023 mg/ml, effective against the DENV-1 virus during the study in HepG2 cells (Ling et al. 2014). In the present, the non-toxic value of *O. sanctum* extracts was noted as 0.031 mg/ml, on C6/36 cells. In our previous study, Kaushik et al. described the secondary compounds on the dengue-2 virus (Kaushik et al. 2018).

The current study was undertaken to test the anti-dengue activity of *Ocimum sanctum* supercritical extract and isolated Eugenol in vitro and in silico and to identify and characterize the isolated compound by different techniques. The inhibitory effect on the dengue virus of *O. sanctum* extract and isolated eugenol was evaluated by the help of qPCR. A qPCR based on the fluorescent hydrolysis probe (Taq Man probe) assay is the latest and most applicable assay. For the results of qPCR, 100 copies of the dengue-2 virus were used for further antiviral assay with the help of a literature study (Lambeth et al. 2005; Zandi et al. 2012; Ramalingam et al. 2018). In present work revealed that the *O. sanctum* supercritical extract, as well as commercial eugenol (99.28%), showed significant antiviral properties against the dengue-2viruses, even treated with a low concentration of 31.25 μg/ml and 15.62 μg/ml, respectively. Many other plants and plants nanoparticle were checked against dengue-2 viruses that played a significant role by inhibiting dengue-2 virus (Kaushik et al. 2018; Sharma et al. 2019). Eugenol (4-allyl-1-hydroxy-2-methoxybenzene) was also tested against other viruses, such as Herpesvirus and Ebola (Lane et al. 2019; Benencia and Courreges 2000).

Molecular docking is beneficial for the synthesis of a better drug for a particular disease. In the present study, the two proteins of dengue NS1 and NS5 were used to study the effect of isolated compounds from the plant extract. NS1 and NS5 are one of the big protein and most drug-targeted domains of the Flavivirus, and they show methyltransferase and RNA-dependent RNA polymerase (RdRp) activities. (WHO 2023). Sonagunalan et al. showed that eugenol (GS-1.23), possesses a good binding affinity to the dengue envelope protein against the dengue-4 target (Sonagunalan et al. 2016). In the present work, eugenol showed good binding energy with both the non-structural dengue proteins (NS1 and NS5) against the dengue-2 serotype. In other studies, the molecular docking against NS1 and NS5 dengue proteins with other compounds showed potential targets of inhibition. The probable mechanistic mechanisms through which the plant extract and isolated chemical demonstrated virucidal action against the DENV-2 strain need to be further investigated. The inhibitory mechanisms of SFE extract of plants can be used to manage dengue. The results of this investigation demonstrated that *Ocimum sanctum* extract and its constituent have virucidal action against the DENV-2 strain of dengue virus. The presence of eugenol in the plant extract may trigger the inactivation of some dengue structural and non-structural proteins, that can be used for their effective control. The maximal binding energy to dengue protein was found using docking to verify the results against the dengue-2. According to molecular docking studies, two NS1 and NS5 are non-structural proteins that can be two potential targets for effective inhibition of the dengue virus. As an antiviral agent, eugenol might serve as a foundation for medication development for the treatment of dengue.

## Data Availability

All data generated or analyzed during this study are included in this article.
